# Too Bright to Focus? Influence of Brightness Illusions and Ambient Light Levels on the Dynamics of Ocular Accommodation

**DOI:** 10.3390/vision9040081

**Published:** 2025-09-30

**Authors:** Antonio Rodán, Angélica Fernández-López, Jesús Vera, Pedro R. Montoro, Beatriz Redondo, Antonio Prieto

**Affiliations:** 1 GIFOA (Grupo de Investigación de Física y Óptica Aplicada), PEXCOG (Experimental Psychology and Cognitive Neuroscience) Research Group, Departamento de Química y Bioquímica, Universidad San Pablo-CEU, CEU Universities, 28668 Boadilla del Monte, Spain; 2 Farmacia López Cubas, Cubas de la Sagra, 28978 Madrid, Spain; angyfl@hotmail.com; 3 CLARO (Clinical and Laboratory Applications of Research in Optometry) Research Group, Department of Optics, Faculty of Sciences, University of Granada, 18071 Granada, Spain; veraj@ugr.es (J.V.); beatrizrc@ugr.es (B.R.); 4 PEXCOG (Experimental Psychology and Cognitive Neuroscience) Research Group, Departamento de Psicología Básica I, Universidad Nacional de Educación a Distancia, 28040 Madrid, Spain; prmontoro@psi.uned.es (P.R.M.); antonioprieto@psi.uned.es (A.P.)

**Keywords:** accommodation, brightness illusion, pupil size, luminance contrast, visual comfort, perceived brightness, chromatic stimuli, digital screen ergonomics, visual fatigue, mesopic lighting

## Abstract

Can brightness illusions modulate ocular accommodation? Previous studies have shown that brightness illusions can influence pupil size as if caused by actual luminance increases. However, their effects on other ocular responses—such as accommodative or focusing dynamics—remain largely unexplored. This study investigates the influence of brightness illusions, under two ambient lighting conditions, on accommodative and pupillary dynamics (physiological responses), and on perceived brightness and visual comfort (subjective responses). Thirty-two young adults with healthy vision viewed four stimulus types (blue bright and non-bright, yellow bright and non-bright) under low- and high-contrast ambient lighting while ocular responses were recorded using a WAM-5500 open-field autorefractor. Brightness and comfort were rated after each session. The results showed that high ambient contrast (mesopic) and brightness illusions increased accommodative variability, while yellow stimuli elicited a greater lag under photopic condition. Pupil size decreased only under mesopic lighting. Perceived brightness was enhanced by brightness illusions and blue color, whereas visual comfort decreased for bright illusions, especially under low light. These findings suggest that ambient lighting and visual stimulus properties modulate both physiological and subjective responses, highlighting the need for dynamic accommodative assessment and visually ergonomic display design to reduce visual fatigue during digital device use.

## 1. Introduction

Accommodation refers to changes in the eye’s dioptric power that allow the visual system to shift focus between objects at different distances, thereby maintaining clear retinal images. Accommodation operates in conjunction with two other ocular physiological responses: pupillary constriction and convergence. Collectively, these three responses form what is known as the accommodation triad or near response [1]. Several physical properties of light directly influence pupillary behavior, including intensity, duration, temporal frequency, spatial extent, peripheral localization, wavelength, and spatial frequency [2,3,4]. These characteristics can affect the latency and amplitude of the pupillary response to varying degrees. Furthermore, some studies have shown that both pupillary responses and subjective perceptions of brightness vary in response to different illusory brightness phenomena. Stimuli that generate a stronger perception of apparent brightness tend to induce greater pupillary constriction and higher subjective brightness ratings [5,6]. These effects also appear to vary with the color of the stimulus [6].

Research examining changes in the accommodative system in response to luminous stimuli remains limited. Accommodative accuracy has been shown to improve when a vertical edge is perceived between two regions with differing luminance contrast (e.g., a red–green bipartite field). Greater luminance contrast resulted in more precise focusing, whereas equiluminance across both hemifields led to reduced focus accuracy [7]. More recently, it has been shown that background–text color combinations can modulate both accommodation and pupillary dynamics. Specifically, blue–red color pairs induced stronger accommodation responses, and positive polarity displays were associated with greater variability in accommodation and smaller pupil sizes [8].

In the field of visual ergonomics, ambient luminance surrounding a display, such as a computer monitor, can reduce stimulus visibility and lead to visual discomfort (i.e., glare) due to luminance differences between the screen and its surroundings. Several authors have proposed that ambient luminance should be equal to or slightly lower than that of the screen itself [9]. Conversely, other studies have shown that excessive ambient lighting can cause reflections and reduce screen visibility, impairing performance and increasing visual fatigue [10,11].

As noted above, various studies have examined how the configuration of visual stimuli, particularly shape, brightness, and color, can trigger various pupillary responses, often linked to perceptual phenomena. However, the accommodative response under such conditions has been far less studied. Given the functional interplay between the accommodation and pupillary systems, it is of interest to explore their dynamics in response to stimuli that induce different levels of brightness and color, under two ambient illumination conditions (high and low), typical of those encountered during electronic device use.

The dynamics of the accommodative system have gained increasing relevance in recent years with the advent of open-field autorefractors. Two key parameters commonly assessed using such instruments are accommodative magnitude and stability (e.g., [12]). These parameters are straightforward to interpret and do not require high temporal resolution. Indeed, with devices like the Grand Seiko WAM-5500 autorefractor, relatively short measurement intervals (30–60 s) have been shown to be sufficient for accurately evaluating both the magnitude and variability of the accommodative response [12].

The aim of this study is to investigate the dynamics of accommodative and pupillary responses under varying brightness illusions and ambient lighting conditions. A better understanding of these ocular responses may provide insights for developing adaptive display technologies designed to reduce visual fatigue during prolonged use of digital devices.

## 2. Materials and Methods

### 2.1. Participants

The minimum sample size required for this within-subject experimental design was based on an a priori calculation using the GPower 3.1 software. Based on the results of a previous study [13] using a similar methodology (Grand Seiko WAM-5500 autorefractor, Rexxam Co., Ltd., Takamatsu-shi, Japan) that found a moderate effect size (Cohen’s d = 0.64) when manipulating participants’ expectations (with a Cohen’s d effect size of 0.64 at 40 cm between placebo and nocebo conditions), the current study requires a sample size of 24 participants. This calculation assumes a more conservative expected effect size of 0.32, with an alpha level of 0.05 and a statistical power of 0.95. All participants included in this study accomplished the following inclusion criteria: (i) between 18 and 35 years of age; (ii) be free of any systemic or ocular disease and no present history of strabismus, amblyopia or refractive surgery; (iii) have normal or corrected-to-normal vision (visual acuity of ≤0 logMAR in each eye), being compensated with soft contact lenses when necessary (contact lens habitual users with at least one year of experience); (iv) have an uncorrected myopia < 0.50 D, hyperopia < 1.00 D, and astigmatism or anisometropia < 1.00 D; (v) no history of refractive surgery or orthokeratology; (vi) presenting normative values for amplitude of accommodation and accommodative facility as indicated by Scheiman and Wick; (vii) report low visual discomfort symptomatology based on the scores of the Conlon visual discomfort survey (<24); and (viii) score < 3 on the Stanford Sleepiness Scale (SSS), which provides a global measure of sleepiness. Participants were instructed to refrain from consuming alcohol and caffeinated beverages for 24 and 12 h, respectively, and to sleep for at least 7 h on the night prior to the experimental session [13].

Although the required sample size based on a priori power analysis was 24 participants, we included 32 individuals to enhance the reliability of the results and better account for interindividual variability. Of the 32 participants recruited (mean age = 25.66 years; SD = 5.37; 23 females), data from one participant were excluded from the analysis of pupillary responses due to poor recording of this variable by the autorefractometer, which resulted in a large number of missing data points.

The experimental protocol follows the guidelines of the Declaration of Helsinki and is approved by the Institutional Review Board (IRB approval: 810/23/101). All participants read and signed an informed consent form prior to their enrollment in the study.

### 2.2. Experimental Setup and Apparatus

**Figure 1:**
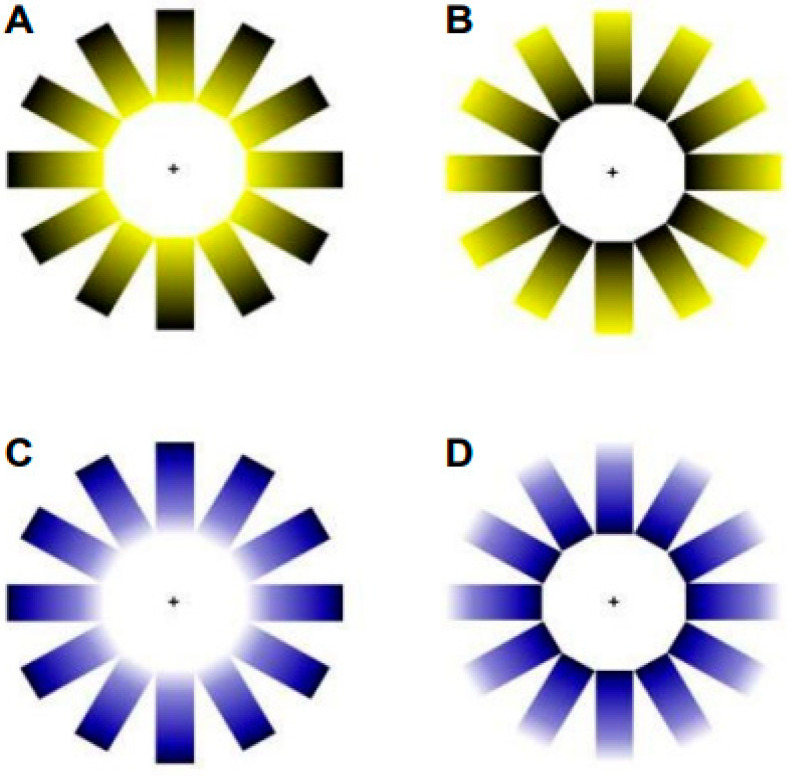
Stimuli used in the experiment. (**A**,**C**) correspond to the two illusions of brightness conditions ((**A**): yellow with brightness; (**C**): blue with brightness). (**B**,**D**) are the two control conditions without illusions of brightness ((**D**) blue without brightness; (**B**) yellow without brightness).

**Figure 2:**
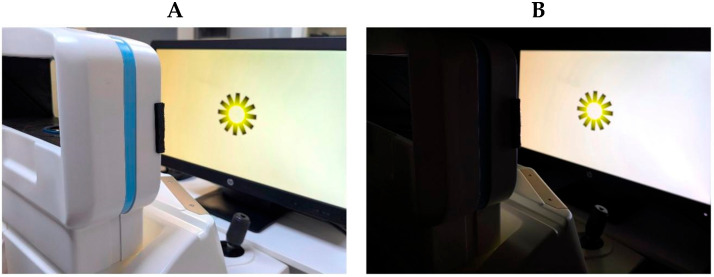
The two ambient lighting conditions in this study: (**A**) low contrast ratio (photopic ambient condition; (**B**) high contrast ratio (mesopic ambient condition).

In this study, we evaluated the effects of (i) the illusion of brightness and color, and (ii) contrast luminance on the dynamics of the accommodative and pupil responses. The experimental manipulation of the main factors is explained below:

(i) **Illusion of brightness and color**. The brightness illusion was induced using an adapted version of the Asahi brightness illusion [5]. Two levels of brightness were presented (with and without the brightness illusion) and two stimulus colors (blue and yellow), resulting in a total of four stimuli (see Figure 1).
(ii) **Contrast luminance**. Room luminance was adjusted relative to the screen brightness to modify the contrast ratio, resulting in two experimental conditions: a **low contrast ratio**, when the room was illuminated (photopic ambient condition); and a **high contrast ratio**, when the room lights were turned off, leaving only ambient light emitted from the screen (mesopic ambient condition) (see Figure 2). According to the guidelines of the Illuminating Engineering Society of North America (IESNA) and the National Board for Industrial and Technical Development (NUTEK), a moderate contrast ratio between the ambient environment and the light source (in this case, the screen) was defined as falling within the range of 3:1 to 10:1. A contrast ratio below 3:1 was classified as a low luminance contrast condition, whereas a ratio above 10:1 was classified as a high luminance contrast condition. Illuminance for the source and the background was obtained using a calibrated luxmeter (model PCE-L335), positioned perpendicularly at both the monitor and the background wall. The illuminance of the source was 430 lx and 40 lx for the photopic and mesopic ambient conditions, respectively. The background illuminance was 295 lx under the photopic ambient condition and 0.05 lx under the mesopic ambient condition. To convert illuminance to luminance, the following formula was applied: L = E × R/π, where *E* represents the measured illuminance and *R* the reflectance factor. A reflectance value of 0.03 was used for the source (matte, non-glossy screen), and 0.8 for the background (matte white wall). Finally, the contrast ratio was calculated as L_source_/L_background_ [14,15]. Accordingly, a contrast ratio of 0.05:1 (low contrast) was obtained for the photopic ambient condition, and 30:1 (high contrast) for the mesopic ambient condition.

A total of eight experimental conditions were conducted (2 (brightness illusion) × 2 (stimulus color) × 2 (luminance contrast)), distributed across two consecutive sessions: one session for the “low luminance contrast ratio condition (photopic ambient condition)” and another for the “high luminance contrast ratio condition (mesopic ambient condition)”. For each of the eight conditions, the magnitude and variability of accommodative and pupillary responses were measured over a 60 s period using the WAM-5500 open-field autorefractor (Grand Seiko Co., Ltd., Hiroshima, Japan) in HI-SPEED mode.

All measurements were performed binocularly, with data recorded from the dominant eye, while participants fixated on an achromatic 2 mm cross at the center of each stimulus to ensure stable fixation and proper gaze alignment. The monitor was tilted at an angle of 105°, and participants’ eyes were aligned slightly above the center of the screen, resulting in a downward viewing angle of 15° relative to the screen center. All sessions were conducted using the same equipment: a 23″ W-LED display (HP ProDisplay P232) with a resolution of 1920 × 1080 pixels, placed at 50 cm from the participants’ eyes. The size of the stimuli was adjusted to the dimensions of the monitor used in the present experiment to subtend the same visual angle as that employed by Suzuki and colleagues [6], who employed a viewing distance of 70 cm, resulting in a visual angle of 11.04°. Accordingly, the physical stimulus size was set to 9.8 cm to match this visual angle. The fixation cross measured 2 mm, subtending a visual angle of 0.23° [6]. The central white area had a diameter of 4.2 cm, and precisely at its geometric center, the achromatic fixation cross measuring 2 mm was presented. For an observer–screen distance of 50 cm, and assuming a nodal distance of the eye of 17 mm (a standard value used to convert visual angle into retinal image size), the retinal sizes, radii, and stimulated retinal regions for each part of the stimulus were as follows: **Central cross (2 mm):** Angular radius (half): 0.115°, retinal radius: 0.034 mm (≈34 µm), falling within the central-most region of the fovea (umbo, fovea pit ≈ 0°); **Central white area (42 mm):** Angular radius: 2.405°, retinal radius: 0.714 mm, covering the foveola and part of the fovea (limit up to ≈5°); **Total stimulus (98 mm):** Angular radius: 5.597°, retinal radius: 1.661 mm, encompassing the fovea and part of the parafovea (according to definitions placing the parafovea at up to ~8° of eccentricity).

### 2.3. Procedure

Prior to the experimental session, an optometric examination was conducted to ensure compliance with the inclusion criteria. When necessary, participants wore their prescribed optical correction during the experiment. The order of the sessions (low and high luminance contrast conditions) and the presentation of the four experimental stimuli within each session were randomized across participants. A 15 min adaptation period was implemented between the two luminance contrast measurement sessions. Additionally, a 3 min rest period was provided between successive measurements, that is, between the presentation of one stimulus and the next.

The Stanford Sleepiness Scale (SSS) was administered at the beginning of the experimental session to assess participants’ levels of alertness/sleepiness [16]. The SSS is a self-reported measure consisting of seven descriptive statements, ranging from 1 (“Feeling active, vital, alert, or wide awake”) to 7 (“No longer fighting sleep, sleep onset soon, having dream-like thoughts”).

Participants were then seated in front of the WAM-5500 autorefractor, using the appropriate chin and forehead rests. Their dominant eye was aligned slightly above the center of the screen, forming a 15° viewing angle relative to the screen center (as described above). First, the monocular refractive state at distance vision was measured using the WAM-5500 in static mode. Following previous recommendations [17], data points exceeding ±3 standard deviations from the mean spherical refraction value will be excluded, as they may result from artifacts such as blinks or recording errors. The accommodative lag –defined as the dioptric difference by which the accommodative demand surpasses the actual accommodative response—was determined by subtracting the average value of the dynamic measurements and the residual refractive error (measured at distance and expressed in spherical equivalent) from the accommodative demand at 50 cm [17,18] (see Equation (1)).

Accommodative lag (D) = Accommodative stimulus − (Residual refractive error at far distance − Accommodative response).
(1)

In addition, the standard deviations of the continuous recordings of accommodative response and pupil size were used as indicators of accommodative variability and pupil size variability, respectively.

The perceived brightness (hereinafter, PB) and visual comfort (hereinafter, VC) associated with each of the four presented stimuli were recorded after the presentation of all four stimuli, in both the low-contrast and high-contrast sessions, while maintaining the respective contrast condition throughout. PB was assessed by displaying all four stimuli simultaneously and asking participants to rate each relative to the white background, which was assigned a reference value of 50. Ratings above 50 indicated greater brightness, while scores below 50 indicated lower brightness compared to the background. VC was also assessed using the same simultaneous presentation of the four stimuli. Participants were asked to indicate the level of VC they experienced while viewing each stimulus. Ratings were given on a scale from 1 to 10, where 1 indicated “very low visual comfort” and 10 indicated “very high visual comfort”.

### 2.4. Statistical Analysis

A randomized repeated-measures design was carried out to assess the effects of brightness illusion, stimulus color, and luminance contrast on ocular accommodation, pupil size, the level of PB, and VC. To this end, separate repeated-measures ANOVAs were conducted, with brightness illusion (with and without illusion of brightness), stimulus color (blue and yellow), and luminance contrast (low and high luminance contrast ratio) as within-subject factors. The dependent variables were the magnitude and variability of the accommodative response and pupil size, as well as PB levels and VC. The level of statistical significance was set at 0.05, and the Holm–Bonferroni correction was applied for multiple comparisons. All statistical analyses were conducted using the JASP 0.19.3 statistical software, and all result-related graphs were generated within this software environment.

## 3. Results

Descriptive values (mean and standard deviation) for the measures of the dynamics of the accommodative response and pupil size at each of the two luminance contrast conditions (low and high contrast ratio) and the four types of stimuli (yellow and blue with brightness illusion; yellow and blue without brightness illusion) are shown in Table 1. For further details, descriptive values of the sample’s demographic data and preliminary tests (visual symptom questionnaire, visual acuity, refraction, and accommodative function) are presented in Table S1 of the Supplementary Material. The Spearman correlation matrix for the continuous variables is presented in Table S2 of the Supplementary Material.

### 3.1. Accommodative Response: Lag and Variability of Accommodation

**Figure 3:**
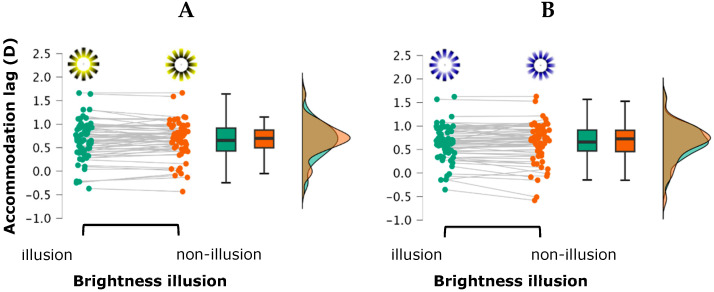
The Raincloud plot illustrates the distribution of accommodation lag across the four stimulus types: (**A**) yellow with and without brightness illusion; (**B**) blue with and without brightness illusion. Each raincloud plot combines a half-violin plot (depicting the probability density of the data), a boxplot (showing the median and interquartile range), and individual data points (jittered for visibility), providing a comprehensive view of both the distribution and variability of the measurements.

A 2 (brightness illusion) × 2 (stimulus color) × (luminance contrast) repeated-measures ANOVA on the variable “lag of accommodation” revealed a statistically significant interaction effect between luminance contrast and stimulus color, *F*(1, 31) = 5.164, *p* = 0.030, η^2^_p_ = 0.143. A greater lag of accommodation was found in the low luminance contrast condition (photopic ambient) for the yellow stimulus compared to the high luminance contrast (mesopic ambient) with the yellow stimulus (mean difference = 0.052, *t* = 1.837, Cohen’s d = 0.131), although this difference did not reach statistical significance after Holm correction (*p*-Holm = 0.379). Similarly, the lag of accommodation was greater in the photopic condition with the yellow stimulus compared to the photopic condition with the blue stimulus (mean difference = 0.041, *t* = 2.289, Cohen’s d = 0.105), but this difference also failed to reach significance after correction (*p*-Holm = 0.174). The rest of the main and interaction effects did not reach statistical significance (all *p*-values > 0.361) (Figure 3). 

**Figure 4:**
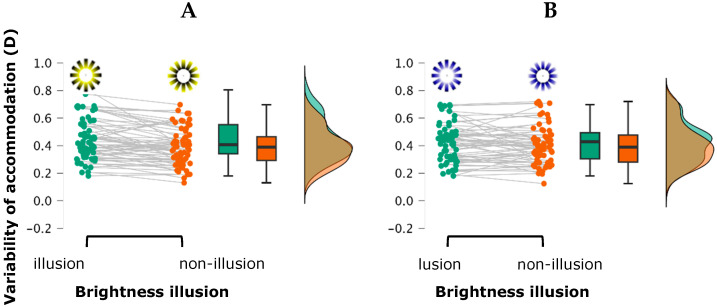
The Raincloud plot illustrates the distribution of variability of accommodation across the four stimulus types: (**A**) yellow with and without brightness illusion; (**B**) blue with and without brightness illusion. Each raincloud plot combines a half-violin plot (depicting the probability density of the data), a boxplot (showing the median and interquartile range), and individual data points (jittered for visibility), providing a comprehensive view of both the distribution and variability of the measurements.

The repeated-measures ANOVA for the variable “variability of accommodation” revealed a significant main effect for the luminance contrast, *F*(1, 31) = 7.697, *p* = 0.009, η^2^_p_ = 0.199, indicating greater variability in the high contrast condition (mesopic ambient) compared to the low contrast condition (photopic ambient). Post hoc analysis confirmed this difference as statistically significant [mean difference = −0.041, *t*(31) = −2.774, *p* = 0.009, *d* = −0.290]. A significant main effect for brightness illusion was also found, *F*(1, 31) = 23.593, *p* < 0.001, η^2^_p_ = 0.432, showing that stimuli with a brightness illusion elicited greater accommodative variability than without the illusion [mean difference = 0.033, *t*(31) = 4.857, *p* < 0.001, *d* = 0.236]. A marginally significant interaction between stimulus color and brightness illusion was observed, *F*(1, 31) = 3.235, *p* = 0.08, η^2^_p_ = 0.094. This effect was driven by a greater accommodative variability in the yellow stimulus with a brightness illusion compared to the yellow stimulus without brightness illusion (mean difference = 0.052, *t*(31) = 4.183, *p*_holm_ = 0.001, Cohen’s *d* = 0.366). No other main effects or interactions reached statistical significance (all *p*-values > 0.183) (Figure 4).

### 3.2. Pupillary Responses: Magnitude and Variability of Pupil Size

**Figure 5:**
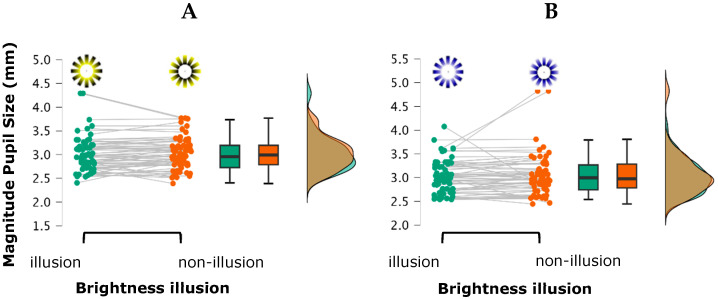
Raincloud plot illustrating the distribution of magnitude of pupil size across the four stimulus types: (**A**) yellow with and without brightness illusion; (**B**) blue with and without brightness illusion. Each raincloud plot combines a half-violin plot (depicting the probability density of the data), a boxplot (showing the median and interquartile range), and individual data points (jittered for visibility), providing a comprehensive view of both the distribution and variability of the measurements.

For the variable “Magnitude of pupil size,” the ANOVA revealed a significant main effect for luminance contrast on pupil size, *F*(1, 30) = 4.917, *p* = 0.034, η^2^_p_ = 0.141, indicating that pupil diameter was significantly larger under the low luminance contrast condition (photopic ambient) compared to the high luminance contrast condition (mesopic ambient) (mean difference = 0.095, *t*(30) = 2.218, *p*_holm_ = 0.034, *d* = 0.251). A marginal effect for stimulus color was also observed, *F*(1, 30) = 2.917, *p* = 0.090, η^2^_p_ = 0.089. This trend reflected a smaller pupil size in response to yellow stimuli compared to blue stimuli (mean difference = −0.029, *t*(30) = −1.708, *p*_holm_ = 0.1, Cohen’s *d* = 0.08). No other main effects or interactions reached statistical significance (all *p*-values > 0.115) (Figure 5). No significant main effects or interactions were found for “pupil size variability” (all *p*-values > 0.236).

### 3.3. Perceived Brightness (PB) and Visual Comfort (VC)

Descriptive statistics (mean and standard deviation) for PB and VC at each of the two luminance contrast conditions (low and high) and the four types of stimuli (yellow and blue with brightness; yellow and blue with no brightness) are shown in Table 2.

**Figure 6:**
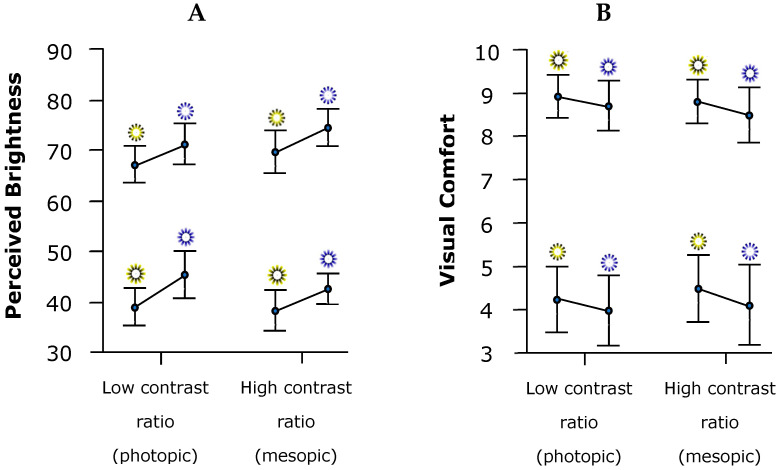
Plot error bar graph (mean, standard deviation, 95% confidence interval), illustrating: (**A**) the PB levels; (**B**) VC for the four types of stimuli as a function of luminance contrast conditions.

For the level of PB, we found statistically significant differences for the main effects of stimulus color (*F*(1, 35) = 13.997, *p* < 0.001, η^2^_p_ = 0.286) and brightness illusion (*F*(1, 35) = 208.124, *p* < 0.001, η^2^_p_ = 0.856), with the blue color and stimulus with bright illusion causing higher levels of PB. The rest of the main and interaction effects did not reach statistical significance (all *p*-values > 0.087) (Figure 6A).

Regarding subjective reports of VC, participants experienced lower levels of VC with the stimulus designed to cause a bright illusion (*F*(1, 35) = 173.545, *p* < 0.001, η^2^_p_ = 0.832). There were no statistically significant differences for the other main or interaction effects (all *p*-values > 0.260) (Figure 6B).

## 4. Discussion

In the present study, we evaluated both physiological responses (accommodation and pupil size) and subjective responses (PB and VC) under two different ambient lighting conditions and in the presence of visual stimuli with varying configurations of color and brightness. Both environmental illumination and the characteristics of the visual stimuli are known to elicit specific physiological responses and subjective perceptions of brightness and VC [5,9]. While pupillary responses have been more extensively studied in relation to color, brightness, and luminance [2,3,4], there is a notable lack of research on how accommodative responses are modulated in these contexts.

### 4.1. Accommodative Responses

Regarding accommodative responses, our findings revealed a significant effect of luminance contrast on accommodation variability, with greater variability observed under high luminance contrast ratio (mesopic ambient lighting) compared to low luminance contrast ratio (photopic ambient lighting). This increased instability may be associated with greater demands on the accommodative system to maintain focus under lower lighting conditions, as suggested by previous studies on the role of luminance in accommodation stability [19]. Moreover, a robust effect of the brightness illusion on accommodative variability was also observed. This may be explained by the fact that brightness illusions involve increased perceptual and cognitive load, which in turn may interfere with the stability of the accommodative system, as previously reported in studies on attentional demand, mental workload, and accommodative responses [20,21]. Several studies have also found that the illusion or sensation of brightness, along with luminance gradients, is closely linked to the perception of apparent depth [22,23,24,25]. In this regard, given the synergy between the vergence and accommodative systems, accommodative fluctuations may be triggered by the depth perception elicited by stimuli involving brightness illusions.

With regard to the magnitude of the accommodative response, a significant interaction between luminance contrast and color was found, with greater lag of accommodation observed under low luminance contrast ratio (photopic condition) and with yellow-colored stimuli. These results are consistent with previous research showing that long-wavelength stimuli (such as yellow or red) tend to exert a greater influence on accommodation than short-wavelength stimuli, even when luminance is matched [26]. Furthermore, the chromatic composition of light can modulate accommodative lag, although the response varies across individuals, likely due to differences in the use of chromatic cues and blur perception [27].

Taken together, these findings suggest that stimuli eliciting stronger brightness illusions and certain colors (i.e., yellow) may modulate the stability of the accommodative response, possibly due to increased perceptual load. With regard to ambient lighting conditions, Chen and colleagues [28] observed that mesopic illumination increased the accommodative lag and variability in both children and adults, particularly in individuals with myopia, compared to photopic illumination. It is worth noting that discrepancies between our results and those of Chen and colleagues may be attributed to differences in the lighting conditions used. Specifically, their photopic condition involved ambient illumination of 500 lux, while the mesopic condition used 10 lux (i.e., dim light). In contrast, our study employed substantially lower background illumination levels for the photopic and mesopic conditions (295 lx and 0.05 lx, respectively), with the monitor serving as a primary light source. This setup resulted in a high luminance contrast ratio under mesopic conditions, which may have contributed to a reduced accommodative lag, especially when yellow stimuli were presented.

In our stimuli, participants fixated a small achromatic central cross, identical across conditions, which served to stabilize fixation and ensure accurate gaze alignment at the stimulus center, whereas the illusion-inducing patterns (central white area and blue/yellow chromatic content) were confined to parafoveal elements of the illusion and represented the true experimental manipulation. These parafoveal patterns were designed to shape the visual context surrounding fixation, rather than to replace the foveal accommodative drive per se. Crucially, because the accommodative and pupillary systems operate in close synergy, the well-established influence of brightness illusions on pupil size provided a strong rationale for testing whether similar contextual effects might also extend to accommodation under the same stimulus conditions. Since accommodation is primarily driven by high-contrast foveal luminance cues, with weaker peripheral contributions that decline with eccentricity, chromatic manipulations outside the fovea were expected to exert only a limited influence on accommodative accuracy [29]. Moreover, any transverse chromatic aberration (TCA) arising from the parafoveal color layout would mainly produce small lateral displacements of colored images in the periphery (fractions of an arcminute near the fovea, increasing with eccentricity), thereby degrading peripheral image quality but not substantially biasing foveally driven accommodation [30]. Consistent with these considerations, our ANOVA revealed at most a marginal main effect of color that did not survive Holm correction, and no pairwise differences between color conditions reached significance, suggesting that color-dependent longitudinal chromatic aberration played only a minor role compared with the luminance and contrast manipulations central to our hypotheses.

### 4.2. Pupillary Responses

Regarding pupil size, no significant effects of color or brightness illusion were observed. However, a significant effect of luminance contrast was found, with larger pupil diameters recorded under a low luminance contrast ratio. This finding appears somewhat counterintuitive, as one would typically expect greater pupil dilation under lower illumination. However, we believe this result may be related to participants’ exposure to the direct light emitted by the monitor displaying the stimuli, which produced a high luminance contrast ratio. For the same luminance level, a light source is perceived as more intense in a dark environment than in a bright one, as described by the Weber–Fechner law. Therefore, under low luminance levels, the retina is more adapted to darkness, and any intense light source is perceived as brighter or even glaring under mesopic conditions. These findings emphasize that the pupil operates not only as an automatic reflex to light but also as an active mechanism responding to visual interpretation and environmental context, adjusting to protect the retina and optimize vision in high-contrast conditions [5,31,32].

In the present study, no effect of the brightness illusion on pupillary responses was observed. This finding contrasts with previous research showing that pupil size is influenced not only by physical luminance but also by PB. For instance, the correlation between the pupillary response and perceived brightness has been shown to be stronger than that with physical luminance, suggesting that subjective brightness perception has a more direct impact on pupil diameter [33]. Similarly, other studies [5,6] demonstrated that the pupil adjusts rapidly according to the intensity of perceived brightness in visual illusions, supporting the notion that pupillary responses reflect the subjective perception of light rather than the mere amount of luminous energy reaching the eye. Further evidence indicates that pupillary constriction is more pronounced when the observer is consciously aware of the bright stimulus, implying that brightness requires conscious processing and that the pupillary response may serve as an objective marker of visual awareness. However, its activation depends on the effectiveness with which the stimulus, or illusion, reaches conscious visual perception [32]. The absence of a pupillary effect in response to brightness illusions in our study may suggest that not all visual illusions are capable of engaging the cortical mechanisms required to elicit a pupillary response, possibly due to differences in illusion strength, experimental context, or the level of awareness achieved by the observer. Although our illusions were inspired by the Asahi stimulus employed by Suzuki and colleagues [6], the salience of our brightness illusion may not have reached the critical threshold required to engage the mechanisms that modulate pupil diameter. It is possible that the perceptual intensity elicited by our stimuli was weaker due to differences in contrast (e.g., the gray background used by those authors versus the white background adopted in our study) or exposure duration (4 s versus 60 s), which could account for the absence of significant pupil size changes in our data.

A marginal effect of color on pupil diameter was observed, with smaller pupil sizes recorded in response to yellow stimuli. These results may align with previous studies suggesting that the pupillary response is influenced not only by physical luminance but also by cognitive and perceptual factors such as color. However, it is worth noting that blue color has been more consistently associated with greater pupillary constriction compared to other colors [6,34]. Also, some studies have reported color-dependent variations in pupillary behavior, reflecting the differential activation of photoreceptors, particularly intrinsically photosensitive retinal ganglion cells (ipRGCs), which are more responsive to blue light and contribute to the sustained pupillary light reflex [35]. The absence of differences in the pupillary response to the blue and yellow stimuli used in the present study may be attributable to both methodological and physiological factors. First, the magnitude of the pupillary response depends on several factors, including stimulus intensity, duration, and area, as well as the participant’s prior state of adaptation and the broader experimental context. If the stimuli do not reach the activation threshold of ipRGCs, or if adaptation conditions are not adequately controlled, the response may be minimal or absent. Moreover, the contributions of rods and cones can mask the specific effects of ipRGCs, particularly under mesopic lighting or when chromatic salience is low [36]. Interindividual variability, chronotype, and circadian factors have also been shown to modulate the spectral sensitivity of the pupil, which may help explain inconsistencies across studies [36]. Finally, the literature suggests that pupillary responses to chromatic stimuli are more robust when using specific chromatic pupillometry protocols or techniques such as silent substitution, which allow for the selective activation of individual photoreceptor types [35,37]. Therefore, the lack of significant effects observed in our study may be attributed to differences in experimental design, stimulus intensity and duration, participants’ adaptation states, or the absence of methodologies that isolate ipRGC contributions.

Although previous studies have reported effects of brightness and color illusions on pupillary responses, we did not observe such effects in our data. We consider these null findings equally relevant, as they contribute to a more balanced representation of the evidence and highlight the natural variability of psychophysiological responses. Negative or divergent results are crucial for refining theoretical models, informing future replications, and reducing publication bias in this field.

### 4.3. Perceived Brightness (PB) and Visual Comfort (VC)

Significant effects were also observed for both PB and VC. A main effect of color revealed that participants rated blue stimuli as subjectively brighter than yellow stimuli. Additionally, a main effect of the brightness illusion showed that stimuli incorporating brightness illusions were perceived as brighter overall. These findings are consistent with previous research on the relationship between color, luminance, and brightness perception [38,39]. Regarding VC, participants reported lower comfort levels in response to stimuli with brightness illusions, suggesting that stimuli perceived as brighter may also be more visually uncomfortable, possibly due to increased perceptual load or visual fatigue. These results are consistent with findings from previous studies showing that screen brightness, particularly under low ambient lighting, can increase perceptual sensitivity and visual fatigue [40,41].

Although our study did not find a significant effect of ambient illumination on perceived brightness or visual discomfort, it is important to note that both subjective discomfort glare and objective veiling glare tend to be exacerbated in darker environments when a localized or point source (such as a screen) emits intense light. As has been suggested, source illuminance, surround illuminance, and ambient illuminance are key factors influencing the subjective ratings of discomfort glare [42]. Moreover, performing certain tasks, such as reading under high screen luminance, has been shown to increase visual fatigue, as evidenced by a reduced blink rate [43]. Prolonged exposure under specific conditions, such as extended use of digital electronic devices, may also lead to other symptoms, including headaches [44].

### 4.4. Implications of the Study

The findings of the present study provide additional evidence on how specific characteristics of the visual environment, such as ambient luminance contrast and the presence of brightness illusions, modulate both physiological responses (accommodation and pupil size) and subjective responses (PB and VC).

From a theoretical standpoint, peripheral responses such as accommodation and pupil size may be influenced by higher-order, top-down perceptual processes, potentially indicating the existence of recurrent feedback mechanisms from the brain to the eyes. In this regard, some studies have suggested that the initial visual input at the retinal level plays a critical role in the generation of illusory experience [45]. Thus, subtle alterations in the early retinal signal could not only trigger the perceptual illusion but may also act as ‘input signals’ upon which cortical mechanisms exert feedback control, modulating both accommodation and pupil diameter according to the subjective interpretation of the stimulus.

From a visual ergonomics perspective, these results reinforce previous recommendations discouraging the use of screens in dark or low ambient light environments [9,11]. Exposure to bright stimuli under such conditions not only increases the subjective perception of glare and visual discomfort but may also exacerbate visual fatigue through physiological mechanisms involving accommodative instability and modulation of pupil size. Thus, mesopic conditions combined with intensely illuminated screens may replicate situations of perceptual glare, a factor that should be carefully considered when designing visual interfaces, work environments, or display systems intended for prolonged use.

Clinically, the assessment of accommodative function under varying lighting conditions and visual stimulus characteristics may be useful in detecting functional instabilities that remain undetected under conventional static testing. In this regard, optometrists should consider incorporating dynamic accommodation tests within simulated environments that reflect real-life screen use scenarios.

Beyond occupational contexts, the present findings also raise concerns about the potential adverse effects of recreational use of electronic devices under low-light conditions, such as those common at night or in dimly lit domestic settings. These seemingly innocuous practices may contribute to the onset of symptoms associated with visual discomfort, eye strain, and even headaches, particularly following prolonged exposure.

Taken together, the findings of this study advocate for a preventive approach, both in the design of visually ergonomic environments and in clinical practice, aimed at minimizing visual fatigue and optimizing visual performance during extended interactions with digital displays.

### 4.5. Limitations and Future Research Directions

Despite the contributions of the present study, certain methodological and conceptual limitations should be acknowledged, as they may have influenced the results and warrant consideration in future research. First, although the experimental design allowed for a controlled evaluation of the interaction between ambient luminance, stimulus color, and the presence of brightness illusions, the generalizability of the findings to real-world settings may be limited. Laboratory conditions, while essential for controlling variables, do not fully replicate the dynamic, multitasking environments typical of real-world occupational or recreational use of digital devices. Moreover, the duration of exposure to visual stimuli was relatively brief (60 s per condition), limiting the extent to which these results can be extrapolated to continuous or prolonged viewing situations, where the cumulative effects of visual fatigue might follow different patterns. It would be advisable for future studies to employ longer tasks, more closely approximating the typical exposure times associated with screen use, in order to more realistically assess the cumulative effects on accommodation, pupillary responses, and visual comfort.

Second, an adapted version of the Asahi brightness illusion was used. However, it is possible that other types of illusions, with different spatial configurations, gradient intensities, or symbolic content, may elicit stronger or more distinct physiological responses, particularly with respect to pupil modulation. The absence of a pupillary effect in response to the brightness illusion may be related to the perceptual salience of the stimuli or to participants’ level of visual awareness. An independent validation of the brightness illusion in a different sample of participants could have provided additional insights into the perceptual salience of the stimuli, and we, therefore, propose this approach as a methodological refinement for future research. Moreover, future studies could incorporate techniques such as silent substitution or chromatic pupillometry to isolate the activation of specific photoreceptor types, such as ipRGCs, to more precisely determine the contribution of chromatic mechanisms to the pupillary response.

Third, although blur was neither intentionally manipulated nor a variable of interest in our study, the stimuli may have induced a perceptual effect of blurriness. Specifically, the spatial arrangement of the elements in the stimulus that generates the brightness illusion could have induced a perception of diffuse contours in the central region, which in turn might have influenced the accommodative response. We consider this a potential limitation of the study and an interesting avenue for future research.

Fourth, a further direction for future studies involves examining interindividual variability and circadian factors—such as chronotype, baseline fatigue, or time of day—which may influence accommodative sensitivity, pupillary responses, and visual comfort. It would also be relevant to explore the interaction of these variables with the nature of the task being performed (e.g., reading, web browsing, dynamic visual tracking), in order to establish more specific ergonomic recommendations.

Finally, this study focused on young adults with healthy vision, excluding individuals with significant refractive errors, accommodative dysfunctions, or moderate to severe visual symptoms. Although this criterion helped minimize potential confounding factors, it limits the generalizability of the findings to broader or more vulnerable populations—such as individuals with progressive myopia, early presbyopia, or a history of digital eye strain—who may be more susceptible to visual fatigue. Future research should explore the cumulative effects of prolonged screen use under high luminance contrast conditions in specific groups, including school-aged children, office workers, myopic and presbyopic individuals, and those prone to visually triggered headaches. Such investigations would contribute to the development of clinically relevant and environmentally adaptive guidelines to prevent discomfort and visual fatigue associated with digital device use.

## 5. Conclusions

This study shows that both ambient luminance contrast and visual stimulus properties, such as brightness illusions and chromatic composition, modulate physiological (accommodative and pupillary) and subjective (brightness and comfort) responses. High luminance contrast increased accommodative variability, while brightness illusions further disrupted accommodative stability, likely due to higher perceptual demand or induced depth cues. Yellow stimuli elicited greater accommodative lag, particularly under photopic conditions. Although pupillary responses were not strongly affected by illusions or color, pupil size decreased under mesopic conditions, possibly reflecting context-sensitive adaptation. Brightness illusions enhanced perceived brightness but reduced visual comfort, especially in low-light environments. These findings highlight the relevance of visual ergonomics in digital screen use, supporting guidelines that discourage bright screen exposure in dark settings. Dynamic accommodative assessment under variable lighting may help detect subtle instabilities not captured by static tests and inform preventive strategies to reduce visual fatigue in everyday and clinical contexts.

## Figures and Tables

**Table 1:** Descriptive (mean ± standard deviation) values of the dynamics of the accommodative response and pupil size.

Contrast Luminance	Color	Bright Illusion	Lag of Accommodation (D)	Variability of Accommodation (D)	Magnitude of Pupil Size (mm)	Variability of Pupil Size (mm)
Low contrast (Photopic condition)	Yellow	Bright	0.68 ± 0.39	0.41 ± 0.14	3.04 ± 0.44	0.79 ± 0.41
		Non-bright	0.70 ± 0.36	0.36 ± 0.13	3.04 ± 0.35	0.82 ± 0.37
	Blue	Bright	0.66 ± 0.37	0.40 ± 0.14	3.05 ± 0.38	0.83 ± 0.44
		Non-bright	0.64 ± 0.41	0.40 ± 0.14	3.11 ± 0.54	0.87 ± 0.44
High contrast (Mesopic condition)	Yellow	Bright	0.64 ± 0.43	0.47 ± 0.16	2.94 ± 0.30	0.82 ± 0.39
		Non-bright	0.64 ± 0.39	0.42 ± 0.14	2.98 ± 0.33	0.81 ± 0.33
	Blue	Bright	0.65 ± 0.37	0.44 ± 0.14	2.98 ± 0.32	0.86 ± 0.38
		Non-bright	0.66 ± 0.43	0.41 ± 0.15	2.99 ± 0.35	0.87 ± 0.42

**Table 2:** Descriptive (mean ± standard deviation) values of PB and VC.

Contrast Luminance	Color	Bright Illusion	PB (0–2100)	VC (0–10)
Low contrast (Photopic condition)	Yellow	Bright	67.17 ± 10.89	4.26 ± 2.24
		Non-bright	38.94 ± 11.16	8.90 ± 1.47
	Blue	Bright	71.97 ± 12.26	3.97 ± 2.38
		Non-bright	45.50 ± 13.71	8.67 ± 1.71
High contrast (Mesopic condition)	Yellow	Bright	69.81 ± 12.69	4.43 ± 2.29
		Non-bright	38.22 ± 12.21	8.81 ± 1.53
	Blue	Bright	74.81 ± 11.08	4.06 ± 2.76
		Non-bright	42.58 ± 9.09	8.49 ± 1.90

## Data Availability

Antonio Rodán had complete access to all study data and is accountable for the integrity of the dataset as well as the accuracy of the analyses performed. Data can be provided upon reasonable request by contacting antonio.rodangonzalez@ceu.es.

## Supplementary Materials

The following supporting information can be downloaded at: https://www.mdpi.com/article/10.3390/vision9040081/s1, Table S1: Descriptive values (mean ± standard deviation) of the sample’s demographic data (age) and preliminary tests: visual symptom questionnaire, visual acuity, refraction, and accommodative function; Table S2: Spearman correlation matrix for continuous variables (N = 32). Positive values (red) and negative values (blue) suggest positive and negative correlation, respectively.
