# Supplementary material for: Too Bright to Focus? Influence of Brightness Illusions and Ambient Light Levels on the Dynamics of Ocular Accommodation
Source: Vision (Basel). 2025 Sep 30;9(4):81. doi: 10.3390/vision9040081 (PMC12551090; doi:10.3390/vision9040081)
Supplement: Supplementary file 1 [file vision-09-00081-s001.zip › vision-3816969-supplementary.pdf]

Table S1. Descriptive values (mean ± standard deviation) of the sample's demographic data (age) and preliminary tests: visual symptom questionnaire, visual acuity, refraction, and accommodative function.

**Variable**	**M ± SD**
Age (years−old)	25.66 ± 5.37
Male (n=9)	26.78 ± 5.59
Female (n=23)	25.22 ± 5.35
Conlon Visual Discomfort Survey	8.73 ± 5.47
Visual Acuity logMAR (far)	
OD	−0.07 ± 0.05
OI	−0.07 ± 0.04
BE	−0.11 ± 0.05
Visual Acuity logMAR (near)	
OD	0.02 ± 0.05
OI	0.01 ± 0.06
BE	−0.01 ± 0.05
Spherical Equivalent (D)	
OD	0.10 ± 0.33
OI	0.10 ± 0.30
Accommodative Amplitude (D)	
OD	10.84 ± 2.59

	OI	10.83 ± 2.59
Monocular Accommodative Facility, MAF (D)		
OD		10.28 ± 3.94
OI		10.94 ± 4.27
Binocular Accommodative Facility, BAF (D)		10.92 ± 3.98

Table S2. Spearman correlation matrix for continuous variables (N = 32). Positive values (red) and negative values (blue) suggest positive and negative correlation, respectively.

		LAG								VA							
0		LC YB	LC YNB	LC BB	LC BNB	HC YB	HC YNB	HC BB	HC BNB	LC YB	LC YNB	LC BB	LC BNB	HC YB	HC YNB	HC BB	HC BNB
LAG	LC YNB	0.907**															
	LC BB	0.930**	0.951**														
	LC BNB	0.866**	0.874**	0.917**													
	HC YB	0.862**	0.849**	0.884**	0.804**												
	HC YNB	0.824**	0.824**	0.809**	0.773**	0.905**											
	HC BB	0.823**	0.815**	0.839**	0.745**	0.927**	0.930**										
	HC BNB	0.866**	0.836**	0.866**	0.783**	0.890**	0.890**	0.925**									
VA	LC YNB	−0.427*	−0.461**	−0.461**	−0.540**	−0.407*	−0.380*	−0.378*	−0.410*								
	LC BB	−0.366*	−0.498**	−0.453**	−0.555**	−0.283	−0.328	−0.214	−0.299	0.741**							
	LC BNB	−0.213	−0.125	−0.214	−0.275	−0.174	−0.109	−0.072	−0.033	0.529**	0.519**						
	HC YB	−0.265	−0.222	−0.272	−0.334	−0.192	−0.178	−0.146	−0.157	0.650**	0.641**	0.805**					
	HC YNB	−.457**	−0.439*	−0.437*	−0.475**	−0.535**	−0.513**	−0.462**	−0.447*	0.703**	0.601**	0.508**	0.484**				
	HC BB	−0.152	−0.235	−0.180	−0.283	−0.273	−0.299	−0.231	−0.119	0.675**	0.623**	0.558**	0.494**	0.792**			
	HC BNB	−0.112	−0.130	−0.091	−0.217	−0.131	−0.215	−0.243	−0.141	0.518**	0.519**	0.508**	0.528**	0.512**	0.632**		
	LC YNB	−0.280	−0.213	−0.217	−0.314	−0.250	−0.208	−0.231	−0.240	0.636**	0.601**	0.638**	0.643**	0.736**	0.678**	0.698**	
MPS	LC YNB	−0.182	−0.201	−0.104	−0.118	−0.147	−0.132	−0.133	−0.244	0.077	−0.040	−0.224	−0.289	0−.031	−0.101	0.010	−0.035
	LC BB	−0.218	−0.251	−0.147	−0.122	−0.216	−0.185	−0.217	−0.316	0.060	−0.061	−0.312	−0.292	−0.067	−0.111	−0.016	−0.032
	LC BNB	−0.050	−0.112	0.024	0.035	−0.072	−0.139	−0.146	−0.122	−0.060	−0.134	−0.266	−0.274	−0.162	−0.042	0.097	−0.078
	HC YB	−0.268	−0.293	−0.176	−0.122	−0.237	−0.239	−0.263	−0.317	0.040	−0.105	−0.273	−0.337	−0.051	−0.114	−0.030	−0.068
	HC YNB	0.106	0.097	0.190	0.152	0.171	0.137	0.196	0.074	−0.087	−0.071	−0.221	−0.225	−0.221	−0.112	−0.099	−0.079
	HC BB	0.004	0.003	0.069	0.096	0.011	−0.019	−0.005	−0.099	−0.127	−0.119	−0.316	−0.321	−0.263	−0.225	−0.079	−0.158
	HC BNB	−0.144	−0.162	−0.074	−0.097	−0.129	−0.109	−0.076	−0.175	−0.010	−0.029	−0.268	−0.264	−0.157	−0.225	−0.043	−0.147
	LC YNB	−0.115	−0.125	−0.036	−0.007	−0.154	−0.161	−0.134	−0.192	−0.057	−.076	−0.256	−0.275	−0.102	−0.066	−0.035	−0.048
VPS	LC YNB	−0.194	−0.070	−0.069	−0.097	−0.089	−0.044	−0.065	−0.098	0.242	0.146	0.221	0.161	0.330	0.283	0.309	0.375*
	LC BB	−0.022	−0.043	0.037	−0.024	0.132	0.056	0.104	0.094	0.252	0.365*	0.145	0.210	0.250	0.306	0.344	0.256
	LC BNB	0.044	0.032	0.093	0.142	0.123	0.081	0.104	0.150	0.208	0.199	0.265	0.238	0.225	0.351*	0.233	0.249
	HC YB	−0.052	−0.045	−0.027	0.032	0.076	0.073	0.067	0.032	0.364*	0.372*	0.387*	0.446*	0.273	0.344	0.258	0.326
	HC YNB	−0.256	−0.231	−0.161	−0.074	−0.193	−0.202	−0.208	−0.196	0.259	0.160	0.019	0.037	0.478**	0.367*	0.195	0.258
	HC BB	−0.054	−0.036	0.008	0.055	−0.039	−0.009	0.031	0.048	0.230	0.122	0.141	0.114	0.359*	0.424*	0.058	0.181
	HC BNB	−0.080	−0.112	−0.029	−0.008	−0.039	−0.018	−0.033	0.052	0.332	0.198	0.201	0.233	0.308	0.379*	0.320	0.191
	LC YNB	−0.085	−0.161	−0.013	0.040	0.023	0.065	0.009	0.043	0.256	0.224	0.170	0.270	0.276	0.259	0.323	0.399*
PB	LC YNB	−0.106	−0.046	−0.085	−0.078	0.088	0.074	0.115	0.015	0.003	0.105	0.002	0.125	−0.168	−0.313	−0.122	0.026
	LC BB	−0.004	0.027	−0.019	0.048	−0.037	−0.154	−0.085	−0.002	−0.291	−0.167	0.094	0.155	−0.117	−0.086	−0.183	−0.139
	LC BNB	0.121	0.092	0.159	0.128	0.138	0.232	0.144	0.054	−0.046	−0.250	−0.398*	−0.118	−0.167	−0.214	−0.106	−0.136
	HC YB	−0.280	−0.270	−0.334	−0.291	−0.255	−0.401*	−0.371*	−0.311	0.043	−0.007	−0.010	0.063	−0.091	−0.119	−0.191	−0.176
	HC YNB	−0.129	−0.193	−0.283	−0.392*	−0.065	0.004	0.021	−0.037	0.264	0.320	0.222	0.241	0.021	−0.033	0.010	0.021
	HC BB	0.074	0.046	0.058	0.172	0.123	−0.003	0.041	0.069	−0.400*	−0.227	−0.130	0.044	−0.246	−0.273	−0.329	−0.206
	HC BNB	0.292	0.268	0.321	0.215	0.292	0.287	0.347	0.258	−0.112	−0.163	−0.323	−0.323	−0.060	−0.104	−0.149	−0.111
	LC YNB	0.097	0.136	0.115	0.188	0.138	−0.013	0.040	0.107	−0.356*	−0.264	−0.078	−0.011	−0.207	−0.161	−0.327	−0.180
VC	LC YNB	−0.056	−0.003	0.065	0.118	−0.094	−0.097	−0.178	−0.073	−0.060	−0.247	−0.013	−0.108	−0.120	−0.091	0.106	−0.034
	LC BB	0.243	0.296	0.262	0.360*	0.170	0.119	0.124	0.121	−0.196	−0.294	−0.148	−0.064	−0.265	−0.200	−0.232	−0.301
	LC BNB	−0.198	−0.185	−0.138	−0.041	−0.155	−0.257	−0.313	−0.215	−0.027	−0.154	0.159	0.079	−0.130	−0.155	0.114	−0.049
	HC YB	−0.068	0.015	0.010	0.190	−0.108	−0.140	−0.228	−0.145	−0.007	−0.311	−0.013	−0.004	−0.003	−0.215	−0.032	−0.057
	HC YNB	0.058	−0.023	0.095	0.196	−0.015	−0.061	−0.117	−0.064	−0.069	−0.207	−0.151	−0.203	−0.120	−0.077	0.001	−0.136
	HC BB	0.292	0.237	0.221	0.328	0.094	0.092	0.030	0.127	−0.122	−0.325	−0.178	−0.127	−0.219	−0.162	−0.171	−0.289
	HC BNB	−0.241	−0.305	−0.289	−0.199	−0.246	−0.333	−0.407*	−0.294	0.001	−0.049	0.056	−0.041	−0.158	−0.147	0.098	−0.160
	LC YNB	0.034	0.158	0.131	0.220	−0.006	0.023	−0.053	−0.015	−0.371*	−0.496**	−0.168	−0.114	−0.269	−0.473**	−0.235	−0.295

		MPS								VPS							
		LC YB	LC YNB	LC BB	LC BNB	HC YB	HC YNB	HC BB	HC BNB	LC YB	LC YNB	LC BB	LC BNB	HC YB	HC YNB	HC BB	HC BNB
MPS	LC BB	0.902**															
	LC BNB	0.796**	0.888**														
	HC YB	0.946**	0.928**	0.870**													
	HC YNB	0.739**	0.786**	0.770**	0.704**												
	HC BB	0.755**	0.845**	0.823**	0.768**	0.911**											
	HC BNB	0.887**	0.873**	0.764**	0.868**	0.783**	0.851**										
	LC YNB	0.796**	0.871**	0.843**	0.831**	0.870**	0.942**	0.862**									
VPS	LC YNB	0.263	0.304	0.261	0.296	0.144	0.145	0.143	0.152								
	LC BB	0.097	0.126	0.166	0.127	−0.005	−0.039	0.026	−0.055	0.715**							
	LC BNB	0.030	0.205	0.318	0.145	0.266	0.213	0.003	0.158	0.667**	0.669**						
	HC YB	−0.048	0.063	0.113	0.012	0.157	0.126	−0.096	0.067	0.616**	0.619**	0.798**					
	HC YNB	0.140	0.248	0.204	0.275	0.112	0.118	0.097	0.190	0.743**	0.608**	0.694**	0.619**				
	HC BB	0.008	0.133	0.162	0.113	0.180	0.103	−0.035	0.151	0.711**	0.525**	0.747**	0.734**	0.851**			
	HC BNB	0.062	0.197	0.262	0.155	0.097	0.059	0.103	0.069	0.691**	0.630**	0.783**	0.693**	0.771**	0.752**		
	LC YNB	0.137	0.290	0.325	0.251	0.174	0.117	0.139	0.160	0.584**	0.611**	0.745**	0.657**	0.679**	0.588**	0.763**	
PB	LC YNB	0.029	−0.102	−0.165	−0.010	−0.114	−0.078	0.017	−0.095	0.090	0.184	−0.180	0.002	−0.124	−0.140	−0.194	−0.024
	LC BB	−0.430*	−0.436*	−0.304	−0.363*	−0.273	−0.291	−0.362*	−0.265	−0.303	−0.183	−0.029	−0.123	−0.208	−0.219	−0.318	−0.219
	LC BNB	0.390*	0.356*	0.287	0.321	0.352*	0.267	0.365*	0.292	0.130	0.051	−0.083	0.040	0.108	0.149	0.056	0.222
	HC YB	−0.238	−0.215	−0.148	−0.162	−0.219	−0.137	−0.256	−0.196	−0.395*	−0.192	−0.108	−0.129	−0.316	−0.365*	−0.320	−0.343
	HC YNB	0.080	−0.026	−0.102	−0.044	−0.043	−0.078	0.093	−0.123	0.056	0.179	−0.058	0.081	−0.223	−0.132	−0.005	−0.027
	HC BB	−0.496**	−0.465**	−0.352*	−0.417*	−0.347	−0.384*	−0.507**	−0.395*	−0.357*	−0.188	−0.065	−0.141	−0.225	−0.250	−0.366*	−0.096
	HC BNB	.0286	0.168	0.130	0.145	0.423*	0.269	0.218	0.216	−0.181	−0.195	−0.196	−0.191	−0.154	−0.043	−0.124	−0.129
	LC YNB	−0.619**	−0.520**	−0.372*	−0.492**	−0.351*	−0.380*	−0.623**	−0.401*	−0.341	−0.173	0.033	−0.059	−0.165	−0.113	−0.319	−0.159
VC	LC YNB	0.091	0.128	0.243	0.207	0.159	0.231	0.145	0.209	−0.130	−0.337	0.012	−0.039	−0.033	−0.072	0.013	0.070
	LC BB	−0.292	−0.281	−0.203	−0.243	−0.300	−0.248	−0.255	−0.287	−0.216	0.024	−0.120	−0.057	−0.196	−0.120	−0.182	−0.274
	LC BNB	0.149	0.055	0.201	0.262	−0.027	0.050	0.079	0.077	−0.239	−0.177	−0.100	−0.109	−0.135	−0.283	−0.226	−0.015
	HC YB	−0.032	−0.081	0.001	0.063	−0.205	−0.059	−0.057	−0.072	−0.190	−0.275	−0.119	−0.077	−0.098	−0.204	−0.094	−0.006
	HC YNB	0.143	0.142	0.257	0.256	0.108	0.151	0.086	0.193	−0.293	−0.226	−0.065	−0.041	−0.022	−0.066	−0.165	0.074
	HC BB	−0.165	−0.253	−0.161	−0.166	−0.334	−0.270	−0.190	−0.289	−0.463**	−0.225	−0.332	−0.321	−0.405*	−0.407*	−0.253	−0.348
	HC BNB	0.259	0.128	0.285	0.338	−0.026	0.111	0.158	0.123	−0.313	−0.171	−0.257	−0.159	−0.263	−0.391*	−0.268	−0.193
	LC YNB	−0.184	−0.189	−0.159	−0.108	−0.239	−0.167	−0.082	−0.164	−0.278	−0.232	−0.282	−0.213	−0.198	−0.263	−0.262	−0.125

		PB								VC						
		LC YB	LC YNB	LC BB	LC BNB	HC YB	HC YNB	HC BB	HC BNB	LC YB	LC YNB	LC BB	LC BNB	HC YB	HC YNB	HC BB
PB	LC BB	−0.022														
	LC BNB	0.081	−0.193													
	HC YB	0.043	0.644**	−0.331												
	HC YNB	0.538**	−0.159	0.078	0.118											
	HC BB	0.105	0.822**	−0.111	0.496**	−0.189										
	HC BNB	0.006	−0.366*	0.428*	−0.183	0.198	−0.277									
	LC YNB	−0.081	0.759**	−0.278	0.599**	−0.291	0.863**	−0.221								
VC	LC YNB	−0.514**	0.001	0.022	0.020	−0.515**	−0.035	0.001	0.078							
	LC BB	−0.066	0.280	0.019	0.243	−0.251	0.315	−0.086	0.448*	0.140						
	LC BNB	−0.042	0.288	−0.120	0.302	−0.308	0.234	−0.382*	0.182	0.505**	0.139					
	HC YB	0.027	0.030	−0.061	0.179	−0.130	0.172	0.041	0.181	0.511**	0.391*	0.434*				
	HC YNB	−0.379*	0.031	0.190	−0.008	−0.502**	0.124	−0.026	0.172	0.692**	0.318	0.611**	0.382*			
	HC BB	−0.058	0.169	−0.060	0.293	−0.122	0.255	0.059	0.270	0.177	0.750**	0.255	0.581**	0.300		
	HC BNB	0.034	0.039	−0.171	0.343	0.019	0.001	−0.186	−0.021	0.352*	0.140	0.800**	0.456**	0.451**	0.393*	
	LC YNB	−0.083	0.243	0.194	0.108	−0.198	0.306	0.017	0.362*	0.487**	0.593**	0.365*	0.625**	0.452**	0.488**	0.284

LAG: lag of accommodation; **VA:** Variability of accommodation; **MPS:** Magnitude of pupil size; **VPS:** Variability of pupil size; **PB:** Perceived brightness; **VC:** Visual comfort; **LC:** Low contrast; **HC:** High contrast; **YB:** Yellow bright; **YNB:** Yellow non−bright; **BB:** Blue bright; **BNB:** Blue non−bright.
*p < .05 **p < .01 (bilateral).
